# Recurrent Acute Pancreatitis Caused by Mucin-Producing Liver Metastases

**DOI:** 10.5334/jbsr.1440

**Published:** 2018-01-31

**Authors:** Anna Evdokimova, Stanislas Laurent, Laurent Dorthu

**Affiliations:** 1 University of Liege, BE; 2 CHR Verviers, BE

**Keywords:** Pancreatitis, Colorectal cancer, Mucinous, Mucobilia, Liver metastases

## Abstract

We present an atypical case of a 69-year-old woman with recurrent episodes of acute pancreatitis and a background history of mucinous colorectal adenocarcinoma. It is emphasized that in the framework of biliary malignant obstruction, modern non-invasive techniques like Magnetic Resonance Imaging (MRI) with hepatospecific contrast agent and Positron Emission Tomography (PET) may help establish the specific cause of the obstruction before any biliary decompression procedure is carried out. This strategy has the potential to prevent from more important tumour spread and provide a greater chance for curative surgery when applicable.

## Introduction

Mucobilia is an abnormal secretion and accumulation of abundant mucus within the biliary duct. It can be the result of biliary mucinosis, biliary papillomatosis, mucin-producing cholangiocarcinoma, cystadenoma, cystadenocarcinoma, or a metastatic mucinous tumour. Around 90% of colorectal cancers are adenocarcinomas. Less frequent (10%) subtypes are mucinous adenocarcinoma and Signet-ring cell carcinoma. These tumours have lower survival rates than the classical adenocarcinomas, as they are often discovered at more advanced stages, including liver metastases [1]. The subsequent biliary obstruction management is often palliative and based on endoscopic retrograde cholangiopancreatography (ERCP). Biliary obstruction from mucobilia caused by liver metastases is an exceptional occurrence that challenges this management, as surgery may remain curative if the mucin-producing metastases can be identified [2]. In this article, we present the case of a 69-year-old woman with recurrent episodes of acute pancreatitis caused by liver metastases from mucinous colorectal adenocarcinoma depicted non-invasively by hepatospecific contrast-enhanced Magnetic Resonance Imaging (MRI).

## Case Report

A 69-year-old woman who has been treated for a colorectal cancer suffered recurrent episodes of acute pancreatitis seven years later. She was initially treated by rectosigmoidectomy, for which further analyses revealed a T2 adenocarcinoma with mucinous differentiation, no distant metastasis, and a N2 nodal stage, leading to a complimentary chemotherapy scheme based on Bevacizumab and FOLFIRI. Follow-up revealed a 3 cm liver metastasis four years later, which required bisegmentectomy (segments VI and VII) with a complete excision of the mass and a new scheme of chemotherapy with Oxaliplatine, Fluorouracil and Folinic acid.

**Figure 1:**
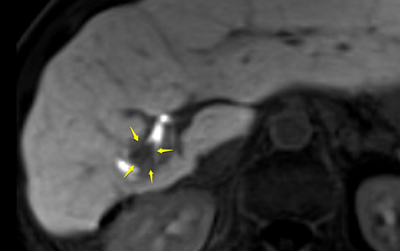
Hepatobiliary contrast (Gd-EOB-DTPA) MRI in the axial plane showed a nodular lesion in the right liver lobe, with low signal intensity compared with the remaining liver, associated with a filling defect in the adjacent bile duct (arrows).

**Figure 2:**
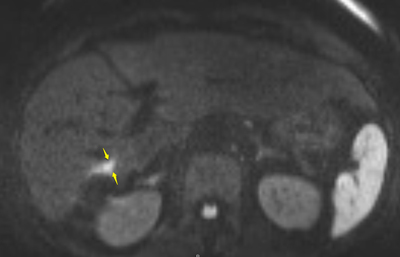
Diffusion-weighted MRI at the same level than Figure 1 showed diffusion restriction in the nodule and the bile duct, characterized by a marked high signal intensity (b-factor value = 800 s/mm2).

Six years after the initial diagnosis, she was admitted with postprandial epigastralgia and elevated levels of lipase (1502, normal range (NR) 5–60UI/L), liver transaminases (AST 546, normal range 10–40UI/L and ALT 417, NR 4–44UI/L), alkaline phosphatase (284, NR 32–104IU) and direct bilirubin (1, NR 0–0.3 mg/dl). Further workup with Sonography, Computed Tomography and Echoendoscopy showed comprehensive dilatation of the bile ducts that contained a dense sludge, but neither lithiasis nor any other cause of obstruction. Endoscopic retrograde cholangiopancreatography (ERCP) revealed bulging of the duodenal papilla and heterogeneous opacification of the bile ducts. The patient was discharged shortly after aspiration of the sludge, sphincterectomy and normalization of the blood tests. The sampled sludge was later identified as mucus containing neoplastic cells. Two months later, after the patient had been readmitted twice for similar episodes and failure to demonstrate any metastasis, MRI with gadoxetic acid disodium (Primovist®), an MRI contrast agent with hepatocyte affinity and biliary excretion was performed, and eventually revealed a filling defect in the liver, a lesion infiltrating the right intrahepatic biliary duct (IHBD) (Figure 1). Its additional properties were restricted diffusion on diffusion-weighted MRI (Figure 2). Two weeks later, a new bisegmentectomy (segments V and VIII) with resection of the right IHBD, saving the hilar plate, were performed. Histopathological examination identified the lesion as a liver mucinous adenocarcinoma metastasis invading the lumen of the bile duct, diffusely replacing the biliary epithelium and infiltrating nerves. Unfortunately, the subsequent care was palliative, as the section was invaded on histology.

## Discussion

This case emphasizes the potential importance of novel imaging strategies in the management of malignant biliary obstruction. First, the diagnosis is usually suggested non-invasively by CT and/or Sonography. The case we presented illustrates pitfalls of this strategy, as diffusion-weighted MRI, a technique of imaging sensitive to cell/microvascular densities, was more sensitive than these non-invasive tests to depict the liver metastasis, as already reported in larger series [3]. The use of a contrast agent with affinity for hepatocytes and biliary excretion further allows for a tridimensional assessment of the biliary tree on MRI, which may provide an advantage over ERCP in case of slightly protruding mural lesions. Lastly, the combination of both diffusion MRI and biliary opacification allows us to differentiate tissular filling defects from other debris, which is of a paramount importance for the diagnosis and management of mucin-producing tumors. Indeed, even though the occurrence of acute pancreatitis caused by liver metastases neoplastic mucus is exceptional [4], this tumour subtype may account for 10% of all colon and rectal adenocarcinomas. Our case also challenges the management of malignant biliary obstruction where the ERCP appearance may call for repeated drainage and even stent placement before the cause is identified. Actually, these procedures are recommended in first intention only in palliative cases [5], as they are invasive and could theoretically aggravate metastatic dissemination. While it remains mandatory to perform a diligent biliary drainage in patients with severe biliary obstruction [6], the placement of a stent may be associated with an inflammatory reaction that would ‘hide’ the causative lesions in the subsequent diagnostic imaging procedures. The present case questions whether patients with low-grade biliary obstruction are not potentially eligible for non-invasive diagnostic assessment of the biliary tree, including advanced techniques like MRI and positron emission tomography to evaluate the cause of obstruction, before ERCP is carried out, if necessary.
